# CTH/MPST double ablation results in enhanced vasorelaxation and reduced blood pressure *via* upregulation of the eNOS/sGC pathway

**DOI:** 10.3389/fphar.2023.1090654

**Published:** 2023-02-13

**Authors:** Antonia Katsouda, Maria Markou, Paraskevas Zampas, Aimilia Varela, Constantinos H. Davos, Valentina Vellecco, Giuseppe Cirino, Mariarosaria Bucci, Andreas Papapetropoulos

**Affiliations:** ^1^ Clinical, Experimental Surgery and Translational Research Center, Biomedical Research Foundation of the Academy of Athens, Athens, Greece; ^2^ Laboratory of Pharmacology, Faculty of Pharmacy, National and Kapodistrian University of Athens, Athens, Greece; ^3^ Department of Pharmacy, School of Medicine and Surgery, University of Naples, Federico II, Naples, Italy

**Keywords:** cystathine γ-lyase, mercaptopyruvate sulfurtransferase, blood pressure, vasorelaxation, nitric oxide synthase, hydrogen sulfide, aorta

## Abstract

Hydrogen sulfide (H_2_S), a gasotransmitter with protective effects in the cardiovascular system, is endogenously generated by three main enzymatic pathways: cystathionine gamma lyase (CTH), cystathionine beta synthase (CBS) and 3-mercaptopyruvate sulfurtransferase (MPST) enzymes. CTH and MPST are the predominant sources of H_2_S in the heart and blood vessels, exhibiting distinct effects in the cardiovascular system. To better understand the impact of H_2_S in cardiovascular homeostasis, we generated a double *Cth/Mpst* knockout (*Cth/Mpst* ^*−/−*^) mouse and characterized its cardiovascular phenotype. CTH/MPST-deficient mice were viable, fertile and exhibited no gross abnormalities. Lack of both CTH and MPST did not affect the levels of CBS and H_2_S-degrading enzymes in the heart and the aorta. *Cth/Mpst* ^*−/−*^ mice also exhibited reduced systolic, diastolic and mean arterial blood pressure, and presented normal left ventricular structure and fraction. Aortic ring relaxation in response to exogenously applied H_2_S was similar between the two genotypes. Interestingly, an enhanced endothelium-dependent relaxation to acetylcholine was observed in mice in which both enzymes were deleted. This paradoxical change was associated with upregulated levels of endothelial nitric oxide synthase (eNOS) and soluble guanylate cyclase (sGC) α1 and β1 subunits and increased NO-donor-induced vasorelaxation. Administration of a NOS-inhibitor, increased mean arterial blood pressure to a similar extent in wild-type and *Cth/Mpst* ^*−/−*^ mice. We conclude that chronic elimination of the two major H_2_S sources in the cardiovascular system, leads to an adaptive upregulation of eNOS/sGC signaling, revealing novel ways through which H_2_S affects the NO/cGMP pathway.

## Introduction

Hydrogen sulfide (H_2_S) is a small endogenously produced molecule with pleiotropic functions (Kimura, 2014; Wallace and Wang, 2015; Cirino et al., 2023). H_2_S along with nitric oxide (NO) and carbon monoxide (CO) are classified as gasotransmitters (Wang, 2002; Paul and Snyder, 2015; Szabo, 2016). Although each gasotransimitter has unique biosynthetic pathways and distinct biological roles, extensive crosstalk between these mediators has been shown to occur at the molecular, cellular and organ levels (Andreadou et al., 2015; Szabo, 2016; Cirino et al., 2017; Kanagy et al., 2017; Szabo, 2017). In the heart, H_2_S promotes cardioprotection; it limits ischemia-reperfusion injury reducing myocardial infarct size and it ameliorates cardiac structure and function in heart failure (Elrod et al., 2007; Bibli et al., 2015; Donnarumma et al., 2017; Li et al., 2018). In the vasculature H_2_S improves endothelial dysfunction, promotes angiogenesis, attenuates atherosclerotic plaque formation and enhances vasorelaxation (Zhao et al., 2001; Papapetropoulos et al., 2009; Suzuki et al., 2011; Bucci et al., 2012; Wang et al., 2015; Bibli et al., 2019). The cardioprotective and angiogenic actions of H_2_S are at least partly mediated by NO (Coletta et al., 2012; Kondo et al., 2013; Xia et al., 2020).

Several sources contribute to H_2_S levels in mammalian tissues. H_2_S can be generated by enzymatic and non-enzymatic reactions; additional H_2_S is released from the consumption of sulfur-containing compounds that are present in the diet and by the gut microbiome (Shen et al., 2013; Kabil and Banerjee, 2014; Filipovic et al., 2018; Yang et al., 2019; Cirino et al., 2023). The main mammalian enzymes that are responsible for H_2_S production are two enzymes of the transulfuration pathway, cystathionine-γ lyase (CTH) and cystathionine-β synthase (CBS), along with 3-mercaptopyruvate sulfurtransferase (MPST), an enzyme of a minor cysteine breakdown pathway (Kabil and Banerjee, 2014; Kimura, 2014; Cirino et al., 2023). The three enzymes use different substrates to generate H_2_S, and have distinct expression profiles and different subcellular distribution. While MPST is equally distributed between the cytosol and the mitochondria, CTH and CBS are predominantly cytosolic under physiological conditions (Fräsdorf et al., 2014; Cirino et al., 2023). It is well known that CTH and MPST are the major sources of H_2_S in the cardiovascular system; CTH and MPST are more abundantly present in both the heart and blood vessels of mice and humans compared to CBS (Peleli et al., 2020).

Although CTH and MPST exhibit some overlapping biological actions, they also exhibit distinct physiological functions. For example, *Cth* ^*−/−*^ mice are hypertensive from a young age and exhibit reduced endothelium-dependent relaxations, while *Mpst* ^*−/−*^ knockout mice have normal responses to vasodilators (Yang et al., 2008; Peleli et al., 2020). In contrast, both CTH and MPST are important for angiogenesis (Papapetropoulos et al., 2009; Coletta et al., 2015). In the heart CTH is cardioprotective; CTH knockout mice exhibited greater infarct sizes after ischemia-reperfusion and a worse phenotype in animal models of heart failure (Kondo et al., 2013; King et al., 2014). On the other hand, MPST knockout mice are protected from cardiac ischemia-perfusion injury, while they exhibit greater deterioration of left ventricular function in heart failure with reduced injection (Peleli et al., 2020; Li et al., 2022).

Given the importance of H_2_S in cardiovascular homeostasis and the importance of CTH and MPST in cardiovascular physiology and disease, we set out to generate and characterize mice lacking both H_2_S-generating enzymes. Surprisingly, the double knockout mice had lower mean arterial blood pressure and exhibited enhanced vasorelaxation due to increased endothelial NO synthase/soluble guanylate cyclase expression. Our findings unravel a novel mechanism of crosstalk between H_2_S and NO.

## Materials and methods

### Mice

C57Bl/6J mice were purchased from the Jakson Laboratory. The CTH knockout (*Cth* ^*−/−*^) and MPST knockout (*Mpst* ^*−/−*^) mice have been previously described (Yang et al., 2008; Nagahara et al., 2013). All animals used for experimentation were bred/housed in individual ventilated cages, under specific pathogen-free, temperature controlled (22°C) and 12 h light/dark cycle conditions in full compliance with the guidelines of the Federation of Laboratory Animal Science Association recommendations in the Laboratory Animal Unit of Biomedical Research Foundation of the Academy of Athens (BRFAA) and allowed free access to diets and water. All studies were performed on male 8–12 week old mice. The lung and kidney from the right side of the experimental animals were used to determine the tissue weight. The left lateral lobe was used to determine the weight of the liver. All experimental procedures reported here were approved by the veterinary authority of the Prefecture of Athens, in accordance with the National Registration (Presidential Decree 56/2013) in harmonization with the European Directive 63/2010.

### Western blotting

Tissues were lyophilized with mortar and pestle and then homogenized in lysis Buffer 150 mM NaCl (Calbiochem, 7760), 1% NP-40 (Sigma-Aldrich, 74,385), 0.5% Na-deoxycholate (AppliChem, A1531,0025), 0.1% SDS (PanReac AppliChem, A2572), 50 mM Tris-HCL, pH = 7.4 (Sigma-Aldrich, T1503), 2 mM EDTA (Merck, 4005) supplemented with a cocktail of protease (PI, Roche, 5,892,970,001) and phosphatase inhibitors (PhoI, Roche, 4906837001). Lysates were centrifuged (13.000 rpm, 15min, 4°C) and the protein concentration in the supernatants was quantified using the DC protein assay (BIO-RAD, 5000116). Concentration was normalized before western blot analysis. Samples were separated on 10% or 12% SDS–PAGE and transferred to a nitrocellulose membrane (Macherey-Nagel; Düren, Germany), after Laemmli buffer containing 4% SDS, 10% β-mercaptoethanol (Sigma-Aldrich, M6250), 20% glycerol (Melford, GI345), 0,004% blue bromophenol (AppliChem, A2331,0025) and 0,125M Tris-HCL, was added. The membranes were blocked [5% milk (PanReac AppliChem, A0830)] and probed with the following antibodies: anti-β-Αctin (Abcam, ab8227), anti-β-Τubulin (Abcam, ab15568), anti-GAPDH (Proteintech, 10494-1-AP), anti-CBS (Proteintech, 14787-1-AP), anti-CTH (Proteintech, 12217-1-AP), anti-MPST (Atlas Antibodies, HPA001240), anti-ETHE1 (Invitrogen, PA5-56040), anti-TST (Proteintech, 16311-1-AP), anti-SQRDL (Proteintech, 17256-1-AP), anti-eNOS (Cell signaling, 32027s), anti-peNOS_s1177_ (Cell signaling, 9571) anti-PKG-I (Cell signaling, 32485s), anti-sGCβ1 (Cayman chemical, 160,897) and, anti-sGCα (Cayman chemical, 160,895). Immunoblots were next processed with anti-rabbit secondary antibody (Merck, AP132P) and visualized using the Western HRP substrate (Merck). Quantification of western blots was performed using ImageJ software (NIH Image, National Institutes of Health, United States).

### Protein persulfidation measurement (Dimedone switch method)

The dimedone switch method was performed as previously described (Zivanovic et al., 2019). In brief, aortas were homogenized in Hens Buffer [50 mM Hepes, 1 mM EDTA, 2% SDS, 0.1 mM neucoproine (Cayman Chemical, 208,745)] supplemented with 1% PI and 20 mM 4-chloro-7-nitrobenzofurazan (NBF-CL, Merck, 10,199-89-0). Lysates were centrifuged (13.000 rpm, 15 min, 4°C) and supernatants were incubated at 37°C for 1 h. Samples were then precipitated by methanol/chloroform precipitation, organic and aqueous layers were aspirated and H_2_O/MeOH/CHCl_3_ was added to the protein pellets and centrifuged. Supernatants were aspirated again, and the pellets were washed (MeOH) and resuspended in 50 mM Hepes containing 1% SDS and 1% PI. Samples were incubated with 50 μΜ cysteine sulfenic acid probe, (DCP-Bio1, Merck, NS1266) for 1 h at 37 °C, precipitated with methanol/chloroform and resuspended in 50 mM Hepes containing 1% SDS and 1% PI. Detection of persulfhydated proteins was achieved using western blood method and a HRP-conjugated anti-biotin specific antibody (Cell Signaling, 5571).

### Blood chemistry and biochemistry

Blood was collected from the orbital venous sinus of mice. Samples were next centrifuged (8,000 rpm, 8 min, 4°C) and serum was isolated. Serum biochemical parameters (alkaline phosphatase (ALP), alanine transaminase (ALT), aspartate aminotransferase (AST), creatine kinase (CK), lactate dehydrogenase (LDH), α-amylase, creatinine, urea, uric acid, albumin, transferrin, ferritin, total-bilirubin, direct-bilirubin, glucose, cholesterol, high-density lipoprotein (HDL) cholesterol, low-density lipoprotein (LDL) cholesterol and triglycerides of WT and *Cth/Mpst* ^*−/−*^ mice were measured.

### Blood pressure measurements

Blood pressure was measured with the non-invasive plethysmography tail-cuff method (Kent Scientific, Torrington, CT, United States). Baseline blood pressure was measured in WT and *Cth/Mpst* ^*−/−*^ mice for 3 days before actually beginning the formal measurements. This is the established training period that allows the mice to acclimatize with the technique and eliminate any stress response. Once, confirmed that all mice showed no signs of stress response, measurements for 2 consecutive days were performed and averaged for the calculation of mean, systolic (SBP) and diastolic blood pressure (DBP); mean arterial blood pressure (MABP) was computed using the equation MABP=(SBP+2DBP)/3. Inhibition of nitric oxide synthase was achieved using *N*_ω_-Nitro-L-arginine methyl ester hydrochloride (L-NAME, N5751, Merck). L-NAME was added in drinking water at a concentration of 0.5 g/L for 10 days.

### Echocardiography

WT and double *Cth/Mpst* knockout mice were anaesthetized using ketamine at a dose of 100 mg/kg by intraperitoneal injection (i.p.) and echocardiographic assessment of left ventricular (LV) function was performed using an ultrasound system (Vivid 7; GE Healthcare) with a 13-MHz linear transducer. Parameters such as heart rate (HR), left ventricular (LV) end-diastolic and end-systolic diameter (LV EDD, LV ESD), LV posterior wall thickness at diastole and systole (PWd, PWs), fractional shortening [FS % = (EDD -ESD)/EDD * 100], ejection fraction [EF% = [(LVEDD^3^-LVESD^3^)/LVEDD^3^]*100] were calculated LV radius to LV posterior wall thickness ratio (r/h) were calculated.

### Evaluation of vascular function

Vascular reactivity was assessed by evaluation of phenylephrine- (PE), acetylcholine- (Ach), the NO donor, DEA-NONOate- and the H_2_S donor, NaHS- induced responses in isolated aortic rings. Mice were anaesthetized with enflurane (5%) and then killed in CO_2_ chamber (70%). The thoracic aorta was rapidly harvested and adherent connective and fat tissue were removed. Aorta was cut in rings of 1–1.5 mm in length and placed in organ baths (3.0 mL) filled with oxygenated (95% O_2_–5% CO_2_) Krebs’ solution (NaCl 118 mM, KCl 4.7 mM, MgCl_2_ 1.2 mM, KH_2_PO_4_ 1.2 mM, CaCl_2_ 2.5 mM, NaHCO_3_ 25 mM and glucose 10.1 mM) and kept at 37°C. The rings were connected to an isometric transducer (Fort 25, World Precision Instruments, 2Biological Instruments, Varese, Italy) associated to PowerLab 8/35 (World Precision Instruments, Biological Instruments, Varese, Italy). The optimal resting tension applied has been previously determined for each mouse strain. The rings were initially stretched until a resting tension of 1.0 g and then were allowed to equilibrate for at least 30 min. During this period, when necessary, the tension was adjusted to 1.0 g, and the bath solution was periodically changed (Mitidieri et al., 2018; Mitidieri et al., 2021). In each set of experiments, rings were firstly challenged with PE (1 μM; Sigma-Aldrich, P16126) until the responses were reproducible. Then PE cumulative concentration-response curve was performed (1 nM–3 µM). In a separate set of experiments, the rings were contracted with PE (1 μM) and, once a plateau was reached, a cumulative concentration-response curve of the following drugs was performed: Acetylcholine (10 nM–30 µM, Sigma-Aldrich, A9101), DEANONOate (10 nM–30 μM, Sigma-Aldrich, D184), N5-(1-Iminoethyl)-L-ornithine dihydrochloride (L-NIO; Sigma-Aldrich I134) and sodium hydrosulfide NaHS (10 nM–3 mM, Sigma-Aldrich, 161,527).

### Statistical analysis

Data are presented as means ± S.E.M. Differences were analyzed using two-tailed unpaired Student’s t-test for comparisons between two-groups. For vascular relaxation studies, differences were analyzed using two-way ANOVA, followed by Bonferroni *post hoc* test. All statistical calculations were made using Graphpad Prism statistical software. Sample sizes are reported in all figure captions. *p* was considered significant when it was less than 0.05.

## Results

### Basic characterization of *Cth/Mpst^−/−^* mice

**FIGURE 1:**
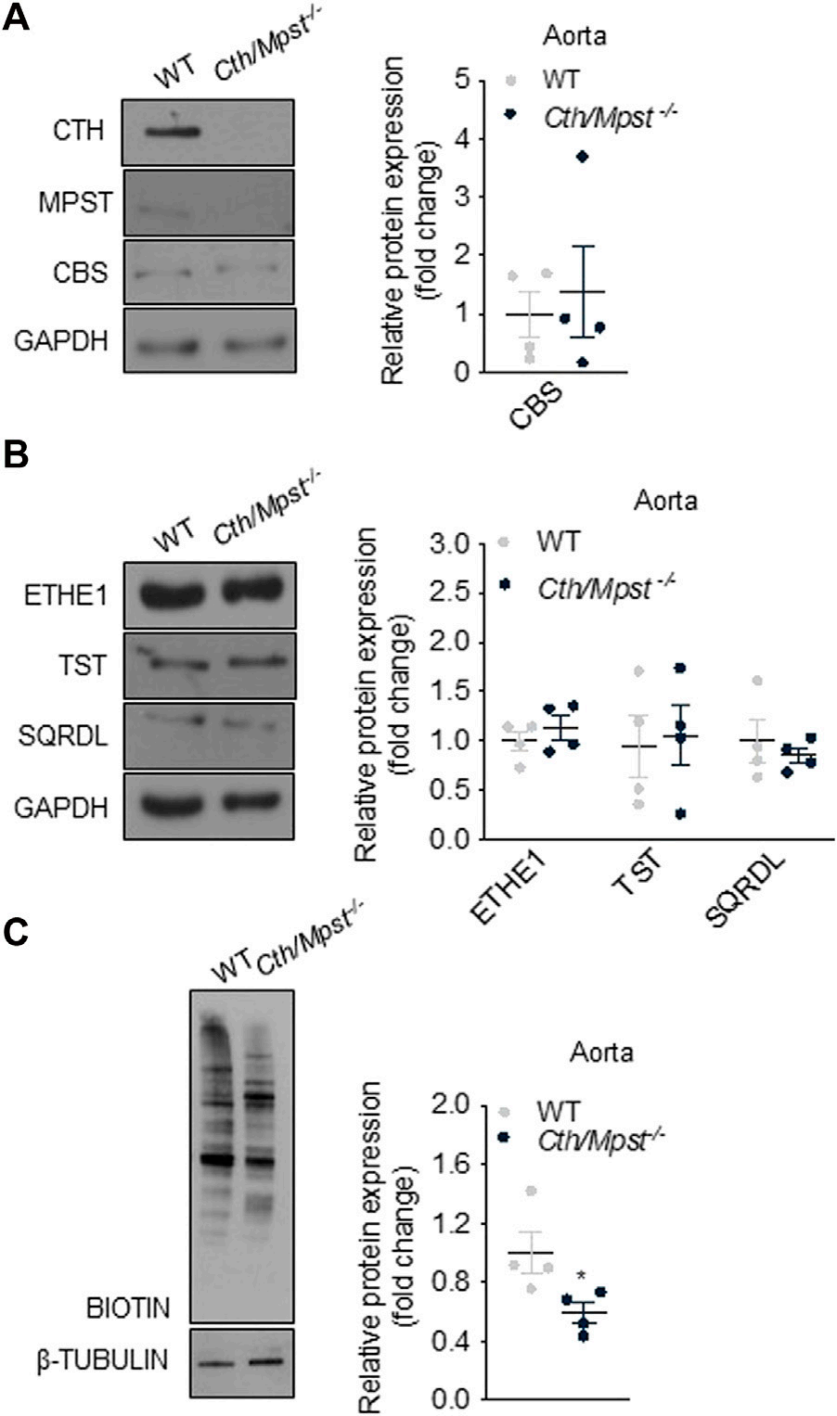
Protein expression of H_2_S-generating and degradation enzymes in aorta of *Cth/Mpst* ^*−/−*^ mice. Proteins were extracted from aorta of WT and double *Cth/Mpst* knockout mice and subjected to SDS-PAGE and western blotting. Representative western blots and quantification of **(A)** MPST, CTH, CBS, **(B)** ETHE1, TST, SQRDL and **(C)** protein persulfidation levels in aorta. Protein expression is presented as ratio over WT group. Data were normalized to GAPDH or β-ΤUBULIN and presented as means ± S.E.M. *N* = 4 mice per group.

**FIGURE 2:**
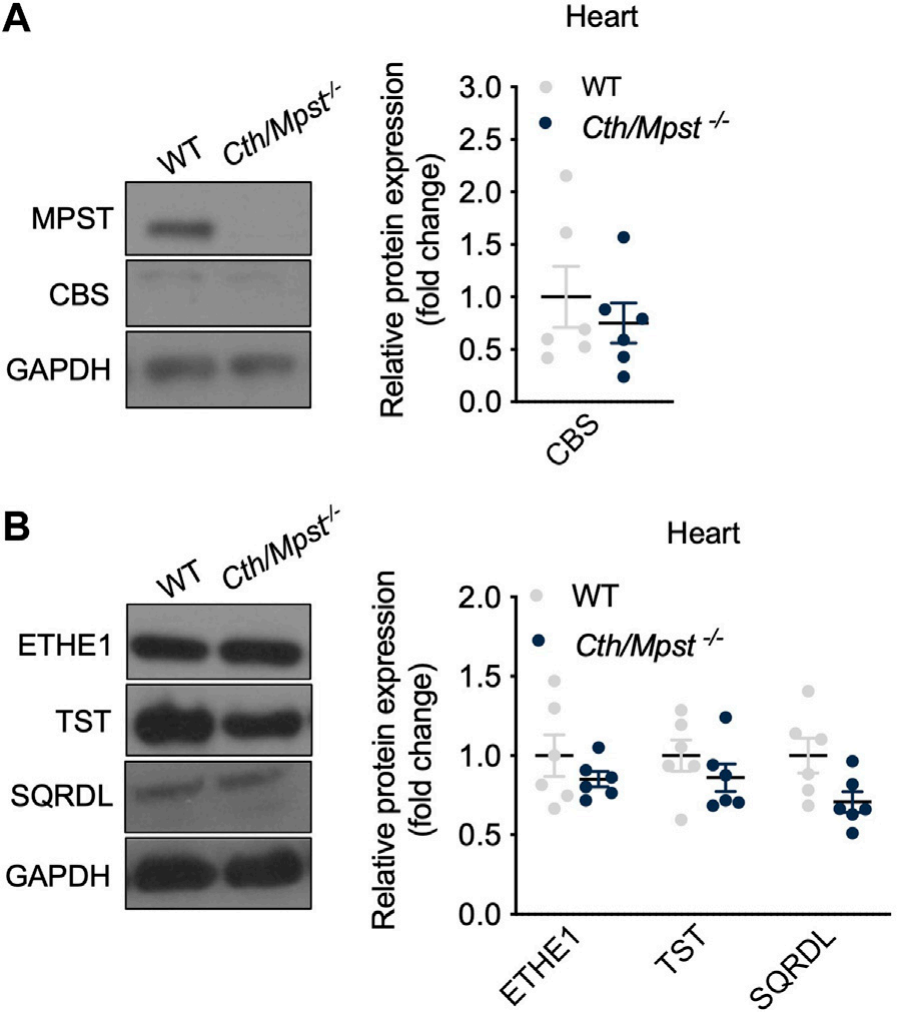
*Cth/Mpst* double deletion does not affect the expression of CBS and sulfide-metabolism enzymes in heart. WT and *Cth/Mpst* ^*−/−*^ mice were sacrificied, proteins were extracted from heart tissues and enzymes leves were determined by western blot. Representative western blots and quantification of **(A)** MPST, CTH, CBS and **(B)** ETHE1, TST, SQRDL levels in heart. Protein expression is presented as ratio over WT group. Data were normalized to GAPDH and presented as means ± S.E.M. *N* = 6 mice per group.

Mice lacking both *Cth* and *Mpst* were generated by crossing *Cth* ^−/−^ and *Mpst* ^−/−^ mice to homozygosity. Lack of CTH and MPST was confirmed in the aorta of *Cth/Mpst* ^*−/−*^ animals at the protein level (Figure 1A). To determine if lack of the two H_2_S-producing enzymes leads to a compensatory increase in the remaining H_2_S-producing enzyme, we measured CBS levels. Lack of *Cth* and *Mpst* did not affect CBS expression. Similarly, no changes in the levels of the H_2_S degrading enzymes ethylmalonic encephalopathy 1 protein (ETHE1), thiosulfate sulfurtransferase (TST) and sulfide quinone reductase (SQRLD) were evident in aortic lysates of double knockout mice (Figure 1B). In line with the attenuated CTH and MPST levels, a reduction in the persulfidation of proteins (a footprint of H_2_S concentration) was detected in aorta of *Cth/Mpst* ^−/−^ (Figure 1C). Experiments to measure the levels of CBS and H_2_S-degrading enzymes in the heart revealed that no major changes were noted in this tissue either (Figures 2A, B). As has been reported before (Fu et al., 2012), CTH was not detectable in the hearts of wild-type mice at the protein level. Body weight, as well as heart and lung weight did not differ between the two strains of mice, while we observed an increase in the kidney and liver mass of double knockout animals (Table 1).

**TABLE 1:** Body and organs weight of WT and *Cth/Mpst* double knock out mice. Data are presented as means ± S.E.M., *p < 0.05 and **p ≤ 0.01, *N* = 4 mice per group.

	WT	*Cth/Mpst* ^*−/−*^
Body weight (g)	27.4 ± 0.3	28.15 ± 0.7
Heart (mg)	157.8 ± 11.4	161 ± 13.9
Kidney (mg)	160 ± 11.8	211.3 ± 10.7*
Liver (mg)	371.5 ± 40.5	647.5 ± 47.3**
Lung (mg)	126.75 ± 5.2	133.3 ± 5.9

### Blood biochemistry of *Cth/Mpst* ^*−/−*^ mice

**FIGURE 3:**
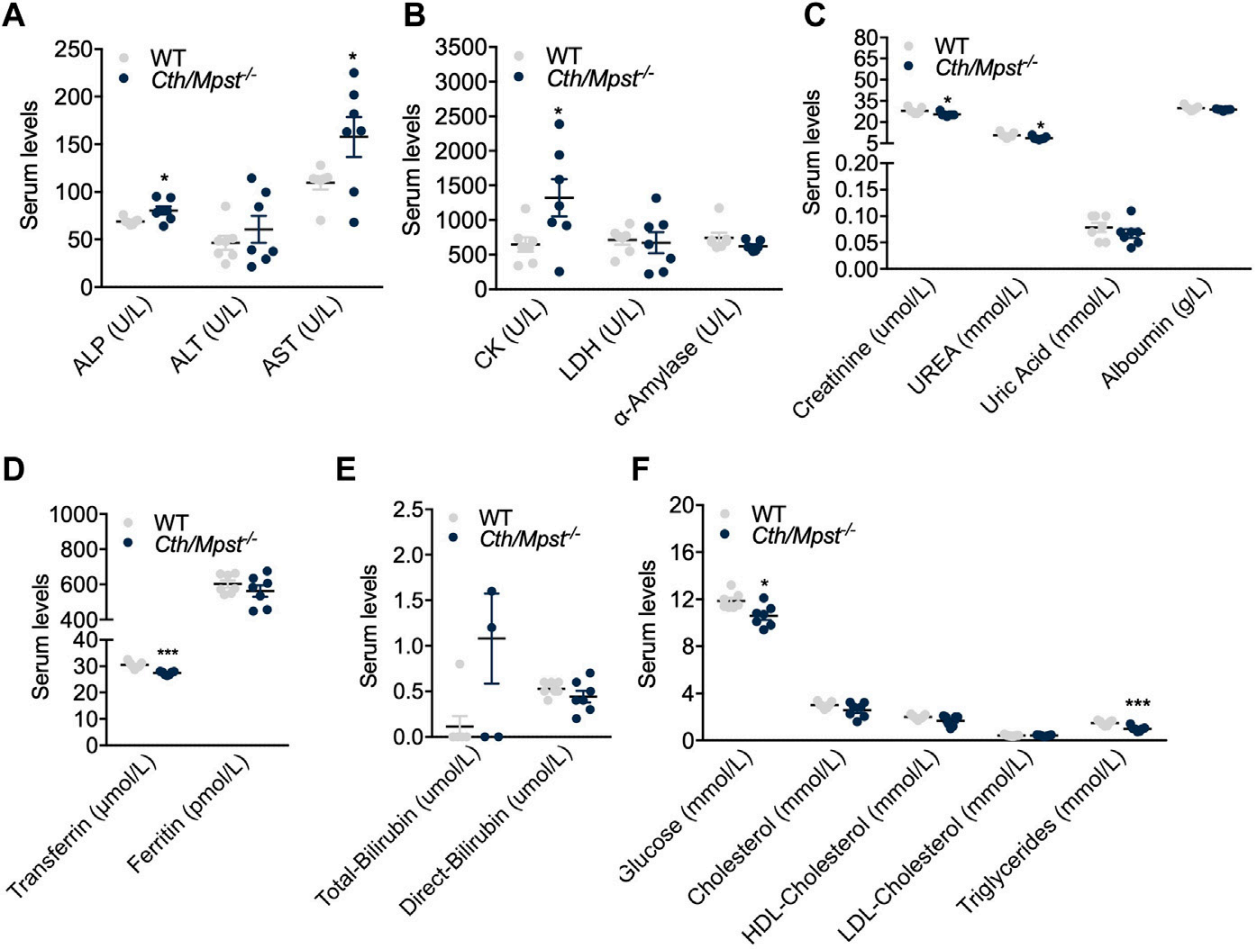
**A**lterations in serum-biochemical parameters after the *Cth/Mpst* double ablation. Serum levels of **(A)** alkaline phosphatase (ALP), alanine transaminase (ALT), aspartate aminotransferase (AST), **(B)** creatine kinase (CK), lactate dehydrogenase (LDH), α-amylase, **(C)** creatinine, urea, uric acid, albumin, **(D)** transferrin, ferritin, **(E)** total-bilirubin, direct-bilirubin, **(F)** glucose, cholesterol, high-density lipoprotein (HDL) cholesterol, low-density lipoprotein (LDL) cholesterol and triglycerides of WT and *Cth/Mpst* ^*−/−*^ mice. Data are presented as means ± S.E.M, *p < 0.05 and ***p ≤ 0,001, *N* = 5–7 mice per group.

We next assessed basic biochemical parameters in the serum of the new mouse strain. Alkaline phosphatase (ALT) and aspartate aminotransferase (AST) were increased in double knockout mice, in line with their grater liver weight observed (Figure 3A). Similarly, double knockout mice had higher serum creatine kinase activity (Figure 3B) and marginally lower creatinine, urea and uric acid levels (Figure 3C). Although these reductions were statistically significant, they were deemed to be of limited or no biological significance. Transferrin (Figure 3D), glucose and triglycerides (Figure 3F) were reduced. Levels of the remaining biochemical parameters tested including lipid levels, bilirubin, ferritin and albumin were not different between the two strains of mice (Figures 3C–F).

### Characterization of basic cardiovascular parameters in *Cth/Mpst* ^*−/−*^ mice

**FIGURE 4:**
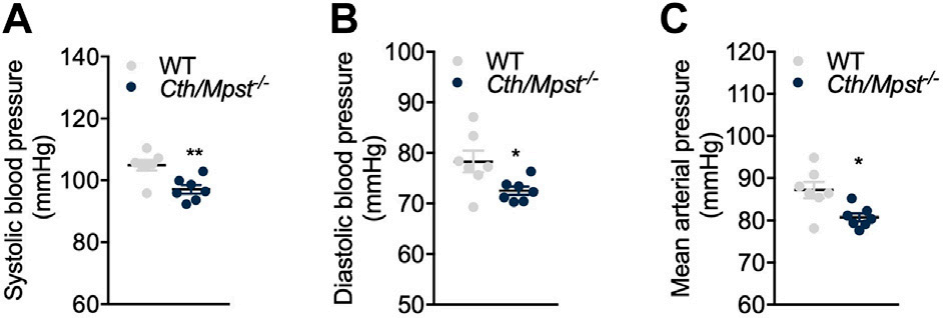
*Cth/Mpst* ^*−/−*^ mice exhibit reduced blood pressure. **(A)** Systolic, **(B)** diastolic and **(C)** mean arterial blood pressure of WT and *Cth/Mpst* ^*−/−*^ mice. Data are presented as means ± S.E.M, *p < 0.05 and **p ≤ 0.01, *N* = 7 mice per group.

**FIGURE 5:**
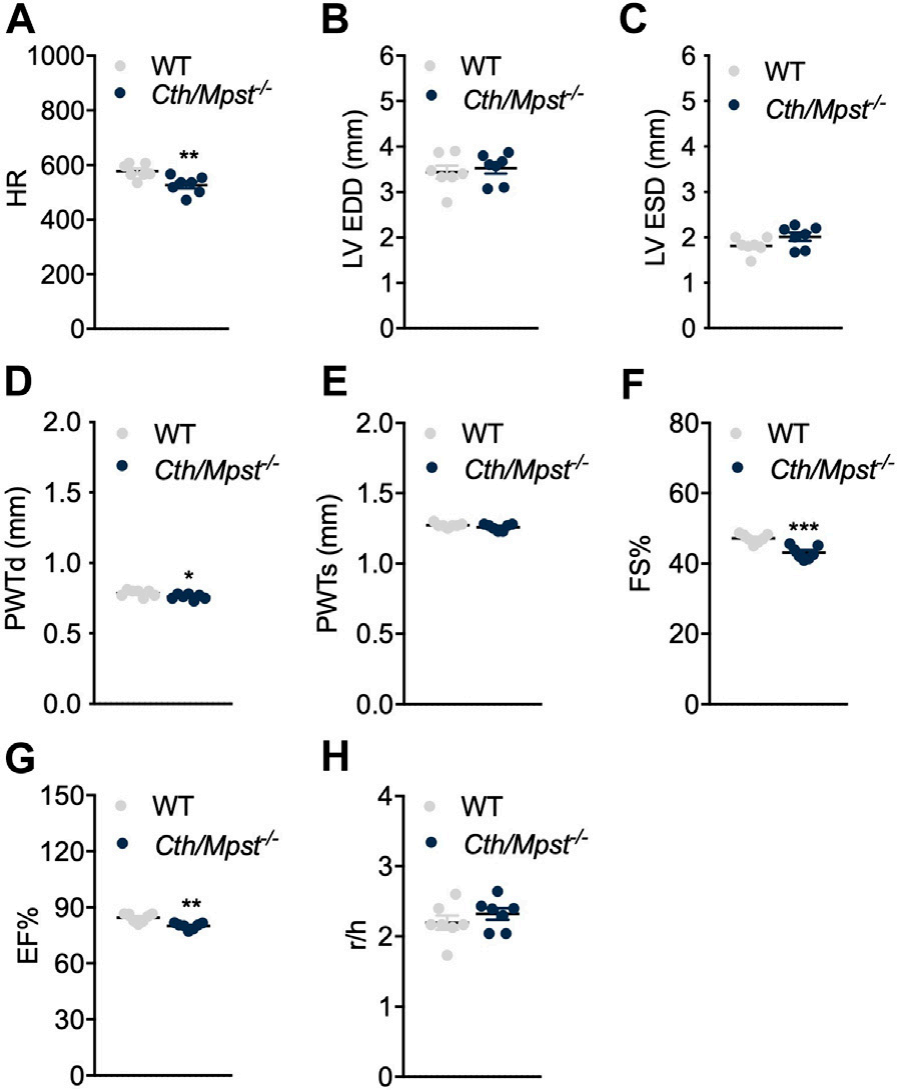
Normal cardiac function parameters after the double *Cth/Mpst* inhibition in mice. **(A)** Heart rate (HR), **(B, C)** left ventricular (LV) end-diastolic and end systolic diameter (LV EDD, LV ESD), **(D, E)** LV posterior wall thickness at diastole and systole (PWd, PWs), **(F)** fractional shortening (FS%), **(G)** ejection fraction (EF) and **(H)** LV radius to LV posterior wall thickness ratio (r/h) analyzed by echocardiography in WT and knockout mice. Data are presented as means ± S.E.M, *p < 0.05, **p ≤ 0.01 and ***p ≤ 0.001, *N* = 7 mice per group.

To evaluate the effect of simultaneous deletion of the two most prominent H_2_S-producing enzymes in the cardiovascular system, blood pressure and cardiac structure and function were measured. Surprisingly, both systolic and diastolic (and therefore mean) arterial blood pressure were lower in double knockout mice (Figures 4A–C). Echocardiography measurements revealed marginal changes in cardiac parameters. Double knockout mice exhibited reduced heart rate (HR, Figure 5A), posterior wall thickness at diastole (PWTd) (Figure 5D), fractional shortening (FS, Figure 5F) and ejection fraction (EF, Figure 5G). The reductions in FS and in EF are too small to be of biological interest. All other parameters measured were similar between the two strains of mice (Figures 5B, C, E, H).

### Vascular responses in *Cth/Mpst* ^*−/−*^ mice

**FIGURE 6:**
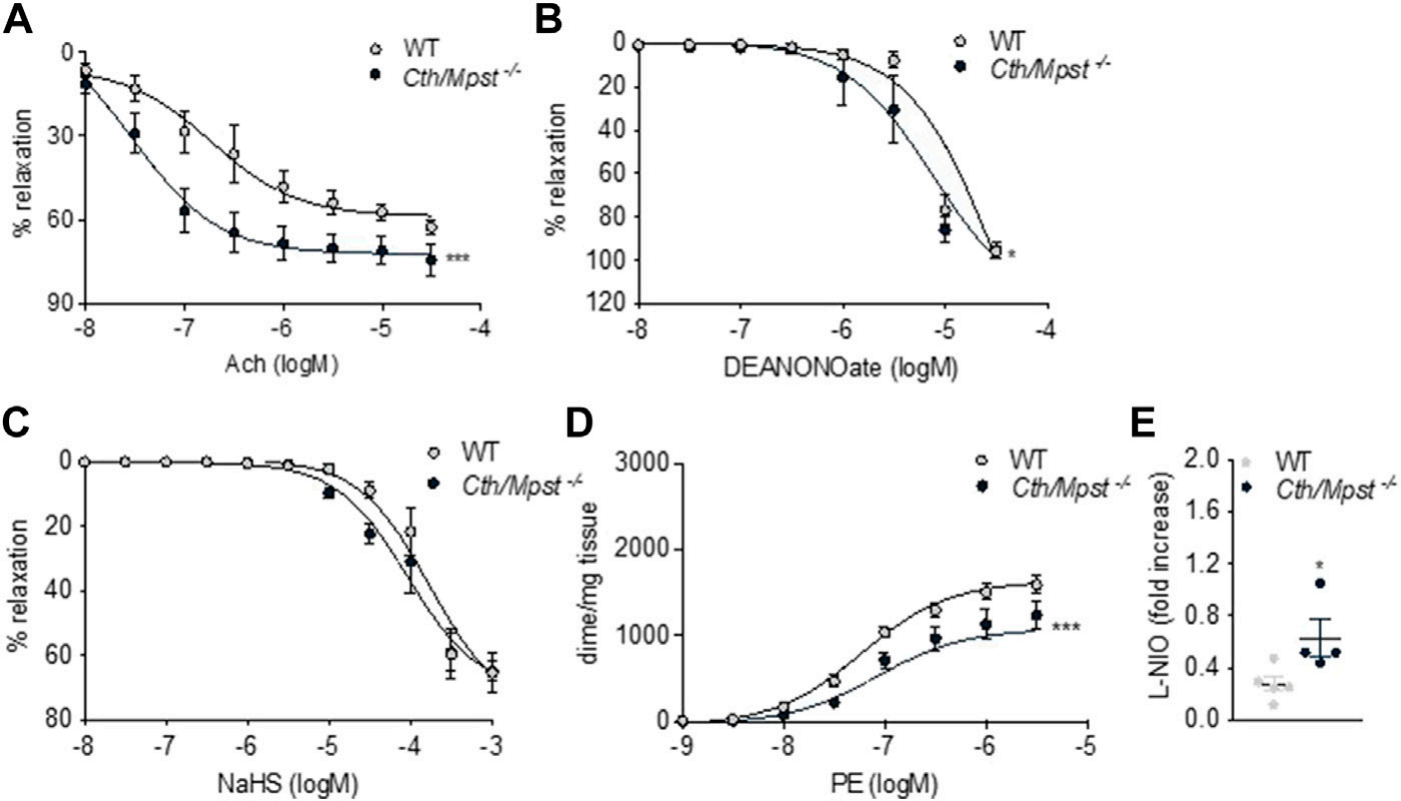
Vascular reactivity measurements of aortic rings from WT and *Cth/Mpst* ^*−/−*^ mice. **(A)** vasodilatatory response to Ach, **(B)** vasodilatory responses to **(C)** the NO donor, DEANONOate and **(D)** the sulfide-donor, NaHS, and **(D)** contractile responses to PE. **(E)** Increase in tension induced by the exposure of PE-pre-contracted aortic rings (300 nM) to L-NIO (10 µM, 20 min). Data are presented as means ± S.E.M, *p < 0.05 and ***p ≤0.001, *N* = 4-6 mice per group.

**FIGURE 7:**
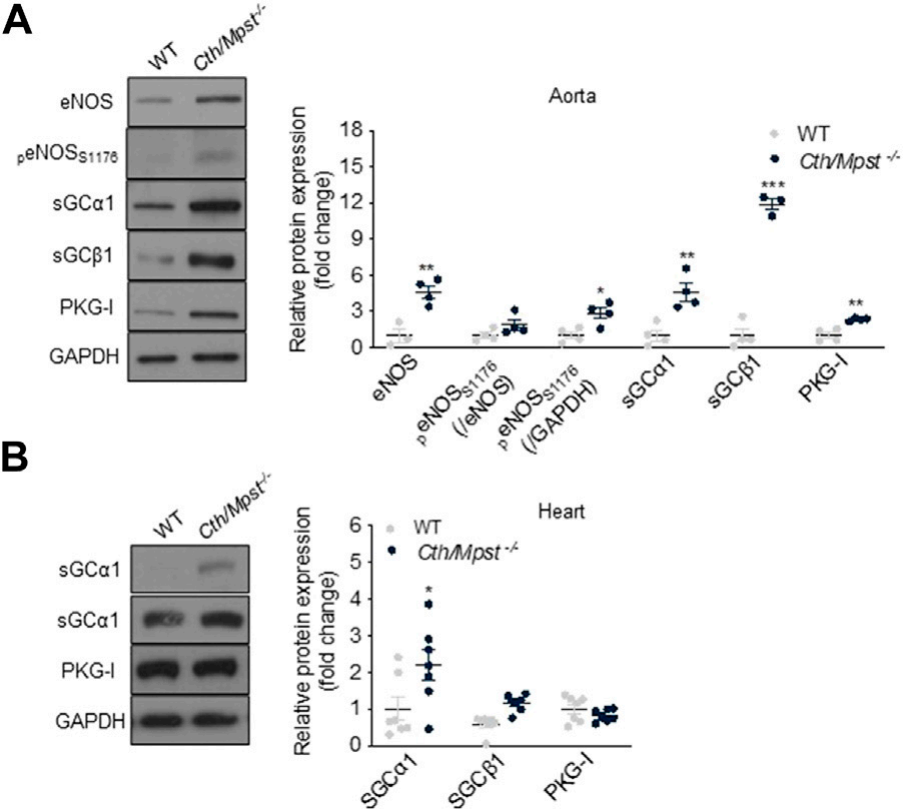
*Cth/Mpst* double ablation results in upregulation of eNOS/sGC signaling in aorta. Representative western blots and quantification of eNOS, _p_eNOS_s1176,_ sGCα1, sGCBβ1 and PKG-Ι protein levels in **(A)** aorta and **(B)** heart protein lysates of WT and *Cth/Mpst* ^*−/−*^ mice. Protein expression is presented as ratio over WT group. Data were normalized to GAPDH or eNOS and presented as means ± S.E.M. *p < 0.05, **p ≤ 0.01 and ***p ≤ 0,001, **(A)** N = 3-4 and **(B)** *N* = 6-7 mice per group.

We next determined the vascular reactivity of aortic rings to vasodilators and vasoconstrictors. In contrast to what would be expected from the literature, but in line with a reduced blood pressure of double knockout mice, relaxation responses to the endothelium-dependent dilator acetylcholine where enhanced in the new mouse strain (Figure 6A). Relaxation to the endothelium-independent NO donor DEANONOate was also slightly enhanced in the double knockout mice (Figure 6B), while responses to the H_2_S donor sodium hydrosulfide were not different between the two strains (Figure 6C). Moreover, phenylephrine caused smaller contractions in the aortic rings of *Cth/Mpst* ^*−/−*^ mice (Figure 6D). In another set of experiments, the selective eNOS inhibitor L-NIO (10 μM) was added on PE-precontracted aortic rings (300 nM) of both WT and *Mpst/Cth^−/−^* mice. After a 20 min incubation, such treatment resulted in a greater increase in tension in *Mpst/Cth^−/−^* indicating an enhanced NO production (Figure 6E). To study the mechanism responsible for the enhanced relaxation seen in the double knockout mice, we determined the expression of endothelial nitric oxide synthase (eNOS), soluble guanylate cyclase (sGC) and cGMP-dependent protein kinase (PKG). Both the α1 and the β1 sGC subunit, as well as eNOS, peNOS_s1177_ and PKG-Ι were more abundant in the aorta of *Cth/Mpst* ^*−/−*^ mice at the protein level (Figure 7A). In contrast, only sGCα1 was increased in the hearts of double knockout mice (Figure 7B).

### Inhibition of NO production restores blood pressure in *Cth/Mpst* ^*−/−*^ mice

**FIGURE 8:**
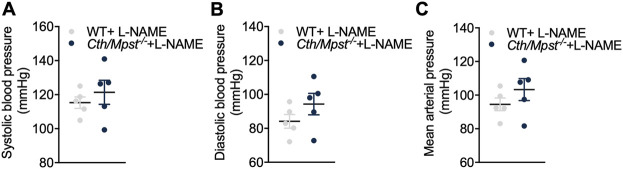
No differences in blood pressure between WT and double *Cth/Mpst* knockout mice after eNOS inhibition. WT and *Cth/Mpst* ^*−/−*^ mice were exposed to eNOS-inhibitor, L-NAME (0.5 g/L in drinking water) for 10 days and blood pressure was measured. **(A)** Systolic, **(B)** diastolic and **(C)** mean arterial blood pressure of WT and *Cth/Mpst* ^*−/−*^ mice after L-NAME administration. Data are presented as means ± S.E.M, *N* = 5 mice per group.

To evaluate the contribution of NO to the reduced blood pressure *in vivo*, we administered the NOS inhibitor L-NAME to mice for 10 days (Figure 8). This treatment lead to elevated blood pressure in both strains of mice; systolic, diastolic and mean arterial blood pressures were similar in wild-type and *Cth/Mpst* ^*−/−*^ mice after L-NAME treatment. These findings suggests that the NO/cGMP pathway is responsible for the lower blood pressure observed in mice lacking both H_2_S-producing enzymes under baseline conditions.

## Discussion

The major findings of our study are that simultaneous global deletion of *Cth* and *Mpst* 1) does not have a substantial impact on cardiac physiology and architecture, 2) results in reduced diastolic and systolic arterial blood pressure, 3) leads to enhanced endothelium-dependent and endothelium-independent vasorelaxation and 4) is linked to an increase in protein levels of eNOS, sGC and PKG-I in the vessel wall.

It should be noted that mice lacking both *Cth* and *Mpst* have been previously generated independently using a CRISPR/Cas9 approach (Akahoshi et al., 2020); however, the cardiovascular phenotype of these mice was not evaluated. The only measurements performed in this strain were basic serum biochemical analytes and amino acid levels, as well as serum, urine and liver levels of compounds related to the general cellular redox state. Serum levels of histidine, cystathionine and citrulline were increased in *Cth*/*Mpst* ^*−/−*^ animals*.* The increase in citrulline is in line with the increased expression of eNOS since citrulline is produced during the conversion of arginine to NO that is catalyzed by eNOS. In addition, lack of *Cth* leads to accumulation of the CTH substrate cystathionine; serum cystathionine levels have been proposed as a biomarker to assess the reduction in CTH activity that is associated with endothelial dysfunction (Bibli et al., 2019). *Cth*/*Mpst* ^*−/−*^ mice were also found to have increased serum homocystathionine and reduced cysteine levels, both of which are expected based on the catalytic activity of CTH (Kabil and Banerjee, 2014; Cirino et al., 2023). In line with the antioxidant properties of CTH and MPST (Nagahara, 2018; Cirino et al., 2023), markers of oxidative stress (oxidized glutathione, total glutathione and thiobarbituric acid-reactive substances) were increased in the serum and liver of *Cth*/*Mpst* ^*−/−*^ mice compared to wild-type control animals. The above observations confirm that lack of the two H_2_S-generation enzymes leads to a pro-oxidant environment *in vivo*.

Additional biochemical measurements in the serum of double knockout mice generated during the course of our study, revealed increased transaminase levels which is in agreement with the observed increase in liver mass. With the exception of creatine kinase which exhibited a two-fold increase and triglycerides which showed a 50% reduction, the remaining analytes measured showed either no difference or minor changes in the range of approximately 10% that although statistically significant in some cases, are of little biological significance.

Hydrogen sulfide levels are determined by both the rate of its production, as well as its degradation rate. Oxidation is the main enzymatic pathway for sulfide elimination and occurs in the mitochondria in two steps (Murphy et al., 2019; Cirino et al., 2023). Sulfide is first oxidized by sulfide quinone oxidoreductase (SQRLD) giving rise to a persulfide. The persulfide is further oxidized to sulfite by persulfide dioxygenase (ETHE1). Sulfite is, in turn, converted to sulfate or thiosulfate by sulfite oxidase (SUOX) and rhodanese (also called thiosulfate transferase, TST), respectively. To evaluate possible compensatory changes in the levels of H_2_S-degrading enzymes in the double knockout mice, we assessed the expression of SQRLD, ETHE1 and TST. None of these was found to be altered in the aorta or in the heart. In agreement with our findings, hepatic TST levels were unchanged in the double knockout mice of the CRISP/Cas9-generated mouse line (Akahoshi et al., 2020). It should be noted that CBS levels were also unchanged in our *Cth*/*Mpst* ^*−/−*^ mouse line.

Cardiac function in *Cth* ^*−/−*^ and *Mpst* ^*−/−*^ has been shown to remain unaffected under baseline conditions (Donnarumma et al., 2017; Peleli et al., 2020; Cirino et al., 2023). To determine if simultaneous deletion of both *Cth* and *Mpst* results in alterations in cardiac physiology, *Cth*/*Mpst* ^*−/−*^ mice were subjected to echocardiography. The most notable feature of these mice that might have physiological significance was a modest decrease in heart rate; the borderline reduction in ejection is likely of minor biological importance. Although no changes in baseline cardiac performance have been documented in *Cth* ^*−/−*^ and *Mpst* ^*−/−*^, both types of mice exhibit a more severe disease phenotype in heart failure and other cardiac pathologies (Kondo et al., 2013; King et al., 2014; Li et al., 2022). Additional studies would be required to evaluate whether double knockout mice exhibit an exacerbated form of cardiac dysfunction in disease models.

To further characterize the cardiovascular phenotype of *Cth/Mpst* ^*−/−*^ mice, we measured arterial blood pressure in awake mice. Although the parental *Cth* ^*−/−*^ mouse strain used to generate the double knockout mice is hypertensive and the *Mpst* ^*−/−*^ normotensive (Yang et al., 2008; Peleli et al., 2020), mice carrying a double *Cth/Mpst* gene deletion have reduced systolic and diastolic blood pressure. This observation is in line with the reduced heart rate in these mice. Notably, when mice were given a NOS inhibitor, blood pressure of both wt and *Cth/Mpst* ^*−/−*^ increased to the same level, suggesting an involvement of NO in the hypotensive response observed in *Cth/Mpst* ^*−/−*^ animals. To determine the vascular reactivity of mice lacking both *Cth and Mpst*, we tested the response of aortic rings to dilating and constricting agents. In agreement to the reduced arterial blood pressure of double knockout mice, contractile responses to the α1 agonist phenylephrine were reduced in rings from *Cth/Mpst* ^*−/−*^ animals. Moreover, we noted significantly greater endothelium-dependent vasorelaxation to Ach and enhanced relaxation to an endothelium-independent NO donor. In contrast to the current observations, acute pharmacological inhibition of H_2_S production reduces endothelium-dependent relaxation and ablation of *Cth* only attenuates endothelium-dependent responses (Yang et al., 2008; Bucci et al., 2010; Xia et al., 2020). It should be noted that our tension measurements were performed in conductance, rather than resistance arteries, which would are important for determining peripheral vascular resistance and blood pressure.

The enhanced dilatory responses to Ach and DEANONOate correlated with increased expression of all of the components of the eNOS/cGMP pathway, namely eNOS, the α1 and β1 subunits of sGC and PKG1. As these changes are tissue-selective occurring only in the aorta (only sGC α1 was increased in the heart), so they are most likely not related to genetic alterations of the double knockout mice. It should be kept in mind that very few stimuli have been shown to increase sGC subunit expression and that to the best of our knowledge there is no known stimulus that can increase the expression of eNOS, sGC and PKG at the same time (Andreopoulos and Papapetropoulos, 2000). Interestingly, H_2_S has been shown to affect mRNA stability and to alter the rate of translation of selected transcripts (Lee et al., 2012; Bibli et al., 2019; Wang et al., 2019). Further experiments would be required to test the mechanism(s) through which lack of CTH and MPST in the vessel wall increases expression of components of the eNOS/cGMP pathway.

In summary, we report that double ablation of *Cth* and *Mpst* results in mice with reduced arterial blood pressure and enhanced responses to vasodilators. Interestingly, the majority of the literature points towards synergistic and/or mutually dependent effects of NO and H_2_S. For example, H_2_S inhibits phosphodiesterase 5 and shifts the sGC redox balance towards ferrous heme to increase its responsiveness to NO(Bucci et al., 2010; Zhou et al., 2016). Also, the angiogenic and cardioprotective responses to H_2_S donors are reduced in eNOS knockout mice, while vasodilation to H_2_S donors is reduced in mice lacking eNOS(Coletta et al., 2012; King et al., 2014; Bibli et al., 2015). Given the interdependence and complementarity in the actions of the two gasotransmitters in the vascular wall, upregulation of the NO arm in the face of complete blockade of the H_2_S production would be homeostatically beneficial. Whether this is of relevance to human pathophysiology remains to be investigated.

## Data Availability

The original contributions presented in the study are included in the article material, further inquiries can be directed to the corresponding author.
